# Clinical outcomes of implants in atrophic ridges augmented with collagenated xenogeneic or autogenous bone: 4-year follow-up of a randomized clinical trial

**DOI:** 10.1590/1807-3107bor-2026.vol40.030

**Published:** 2026-06-22

**Authors:** Giuseppe Alexandre ROMITO, Herbert Horiuti SOARES, Roger NISHYAMA, Marina Clemente CONDE, Frank SCHWARZ, Cristina Cunha VILLAR

**Affiliations:** (a) Universidade de São Paulo – USP, School of Dentistry, Department of Stomatology, São Paulo, SP, Brazil.; (b) Johann Wolfgang Goethe-University Frankfurt, Department of Oral Surgery and Implantology, Carolinum, Frankfurt, Germany.

**Keywords:** Follow-up Studies, Dental Implants, Alveolar Ridge Augmentation, Bone Regeneration, Heterografts

## Abstract

This study aimed to evaluate the long-term outcomes of dental implants placed in alveolar ridges augmented with collagenated xenogeneic bone blocks (CXBB) or autogenous bone blocks (ABB). In this non-interventional study, 20 patients (CXBB: n = 11; ABB: n = 9) from a previous randomized controlled clinical trial were followed for four years. These patients had undergone lateral alveolar ridge augmentation with either CXBB or ABB, with implants placed after a 30-week healing period. The following parameters were assessed: implant survival and success rates, peri-implant clinical parameters, Pink Esthetic Scores (PES), and patient-reported outcome measures (PROMs). Both groups demonstrated high implant survival rates, with only one late failure occurring in the ABB group. There were no statistically significant differences between the CXBB and ABB groups regarding implant survival and success rates (p > 0.05). Peri-implant clinical assessments indicated healthy tissue surrounding the implants in both groups, with no significant differences between CXBB- and ABB-grafted sites. While overall PES scores were similar, the ABB group showed significantly higher scores for soft tissue level and contour (p < 0.05). Patient-reported outcomes, accessed via the OHIP-14 questionnaire, indicated comparable satisfaction levels between the two groups. Both CXBB and ABB groups exhibited comparable long-term outcomes for implant survival and success. Although the ABB group demonstrated slightly better esthetic results, CXBB showed comparable clinical and patient-reported results, suggesting it may be a feasible alternative for staged lateral alveolar ridge augmentation. These findings should, however, be interpreted with caution given the limited number of patients.

## Introduction

The long-term aesthetic and health status of dental implants are closely tied to the quality and quantity of the supporting alveolar bone. After tooth extraction, the alveolar ridge undergoes resorption, leading to significant three-dimensional changes that can compromise implant placement, stability and long-term outcomes.^1-6^ This challenge has led to the widespread adoption of alveolar ridge augmentation techniques, which are crucial for ensuring adequate bone volume for prosthetically driven implant positioning. These techniques can be implemented either in stages, particularly when large lateral alveolar ridge defects prevent the achievement of implant primary stability, or simultaneously with implant placement.^7-9^

Autogenous bone blocks (ABB) have long been regarded as the gold standard for staged lateral ridge augmentation due to their osteogenic, osteoinductive, and osteoconductive properties.^10^ However, the use of ABB is often linked to considerable donor site morbidity and limited availability, which has driven efforts to identify alternative grafting materials.^11^

In recent years, collagenated xenogeneic bone blocks (CXBB) have emerged as a promising alternative to ABB, offering the advantages of ready availability and eliminating the complications associated with donor site harvesting. Preclinical and short-term clinical studies have demonstrated that CXBB can achieve comparable outcomes to ABB in terms of bone gain, implant survival and success.^12-26^ Despite these promising findings, further longitudinal data are needed to confirm the long-term efficacy of CXBB, particularly in comparison to the well-established ABB.

This study extends our previous research, which demonstrated the non-inferiority of CXBB compared to ABB in terms of ridge width gain, implant survival and success rates after one year.^24-26^ In this follow-up, we present a 4-year evaluation of the same cohort, providing an evaluation of the long-term clinical performance of implants placed in ridges augmented with CXBB versus ABB.

## Methods

### Study design

This is a non-interventional, 4-year follow-up study of the original non-inferiority randomized clinical trial, which assessed the gain in linear bone thickness (LBT), measured 2 mm below the alveolar crest, following lateral grafting with CXBB versus ABB.^24^ The study was conducted in accordance with the principles of the 2008 revision of the Helsinki Declaration of 1975 and was approved by the Ethics Committee of the University of São Paulo, School of Dentistry), being conducted from January to July 2024. The study is registered with the German Register of Clinical Trials (DRKS00017137) and follows the STROBE guidelines for reporting observational studies.

### Participants

Participants from the original non-inferiority randomized clinical trial ^24^ were eligible for this follow-up study. The original trial included 64 patients with atrophic alveolar ridges, who underwent lateral bone grafting. Implants were placed in the augmented sites after a 30-week healing period, followed by the delivery of final screw-retained restorations 11 weeks later.^24^

For the 4-year follow-up, the eligibility criteria were as follows:

*Inclusion criteria*:

- Enrollment in the previous randomized clinical trial comparing CXBB and ABB for lateral bone augmentation.^24^
- Ability to comprehend and sign the informed consent form.

*Exclusion criteria*:

- Newly diagnosed systemic conditions affecting bone metabolism.
- Significant trauma to the implant site (e.g., direct impact injuries resulting in soft tissue laceration or fracture of the surrounding alveolar bone).
- Surgical interventions in the implant site.
- Orthodontic treatment in the same arch as the implant.
- Participation in another clinical study that might interfere with the objective of this follow-up study.

All participants provided written informed consent after receiving a comprehensive explanation of the study’s procedures and objectives.

### Augmentation procedures

The augmentation procedures performed by a single experienced clinician (GAR) in the original study have been detailed previously.^24^ In summary, full-thickness mucoperiosteal flaps were raised to expose the alveolar bone. Cortical perforations and bone flattening were performed to enhance vascularization and facilitate graft integration. In the CXBB group, the graft material (CXBB; Geistlich Pharma AG, Wolhusen, Switzerland) was contoured to fit the defect and secured with fixation screws. For the ABB group, bone blocks were harvested from the retromolar area, shaped, and fixed similarly. Both groups received additional deproteinized bovine bone mineral (DBBM) (Geistlich Pharma AG, Wolhusen, Switzerland) along the block edges and were covered with a collagen membrane (CM) (Geistlich Pharma AG, Wolhusen, Switzerland), followed by tension-free submerged healing. Sutures were removed two weeks post-surgery.

### Implant placement and prosthetic procedures

After a 30-week healing period, implants were placed in the augmented sites using a guided approach.^24^ Full-thickness flaps were raised, and the fixation screws were removed prior to implant placement. One bone-level implant (Institut Straumann AG, Basel, Switzerland) was installed per site using 3D-printed drilling templates (Swissmeda, Zürich, Switzerland). Implant length and diameter were selected based on preoperative CBCT data. In cases where additional bone regeneration was necessary, DBBM and CM were applied. Following a 7-week submerged healing period, the implants were exposed, and definitive screw-retained crowns were delivered within four weeks.

### Support care and outcome assessments

During the supportive care, visit frequency was tailored to each patient’s needs, ranging from one to three visits per year. These appointments included comprehensive evaluations of periodontal and peri-implant health, personalized oral hygiene instructions, professional prophylaxis and mechanical debridement as needed.

The 4-year follow-up visits occurred between 48.0 to 67.7 months post-restoration, with a mean follow-up duration of 55.0 months post-restoration and 62.8 months post-implant placement. Implant survival was defined as the presence of the implant in the patient’s mouth at the time of assessment. Early failures were defined as those occurring prior to abutment connection, and late failures as those occurring after abutment connection. Implant success was evaluated according to the criteria established by the Pisa Consensus Conference. ^27^ Additionally, during the 4-year follow-up visit, the following parameters were recorded:

### Clinical measurements

Using a North Carolina periodontal probe, a single trained and calibrated investigator (GAR) collected the following data:

- Modified plaque index (mPI).^28^
- Bleeding on probing (BOP) within 30 seconds after gentle probing.
- Probing depth (PD), measured from the peri-implant mucosal margin to the base of the sulcus/pocket and recorded to the nearest millimeter (mm).
- Position of the peri-implant mucosal margin (MM), measured in mm from the mucosal margin to the implant shoulder, with MM positioned coronal to the shoulders recorded as negative values.^29^
- Probing attachment level (PAL), measured from the base of the sulcus/pocket to the implant shoulder and recorded to the nearest mm.
- Keratinized tissue (KT), measured from the mucogingival line to the peri-implant MM and recorded to the nearest mm.

These clinical parameters were measured at six sites per implant (mesio-buccal, mid-buccal, disto-buccal, mesio-lingual, mid-lingual, disto-lingual), except for KT, which was measured at the mid-buccal site only.

### Pink esthetic score

The esthetic evaluation of the soft tissues surrounding the implants was conducted using the Pink Esthetic Score (PES) ^30^ during the clinical exam by an experienced and calibrated investigator (GAR).

### Radiographic analysis

Periapical radiographs were acquired using phosphor storage plates (KaVo, Biberach, Germany). The captured images were processed with the Scan eXam One software (Dexis, Arlington, United States) and analyzed using the system’s measurement tool, which provides measurements in millimeters. Marginal bone loss was determined by measuring the distance from the implant platform to the first point of bone-to-implant contact on both the mesial and distal aspects. All analyses were performed by a single examiner (HHS) who had been previously trained and calibrated. A total of 20 radiographs were evaluated on two separate occasions, with a 48-hour interval between assessments. The intra-examiner reliability, assessed using the intra-class correlation coefficient (ICC) demonstrated an agreement of 0.912.

### Profilometric and linear outcome measures

Optical impressions were taken at crown loading (baseline) and after 4 year using the Virtuo Vivo Scanner (Dental Wings, Quebec, Canada). The resulting STereoLithography (STL) files were analyzed with Software (Swissmeda, Zürich, Switzerland), where central transversal images were aligned using reference lines. Central transversal images were used to set a vertical reference line parallel to the implant axis, and a horizontal line, perpendicular to the vertical reference line, positioned at the level of the peri-implant mucosal margin (MM) at baseline.^26^ Three additional horizontal lines were positioned 1, 3 and 5 mm apical to this first one (Figure). Tissue thickness (eTT) was measured at 1, 3, and 5 mm below the peri-implant MM, and changes were calculated by comparing baseline and 4-year follow-up values. Mean profilometric changes (PC) were also determined, evaluating the distance between surfaces in the region extending 3–6 mm apically from the peri-implant MM. All analyses were performed by a single examiner (HHS) who had been previously trained and calibrated.

**Figure:**
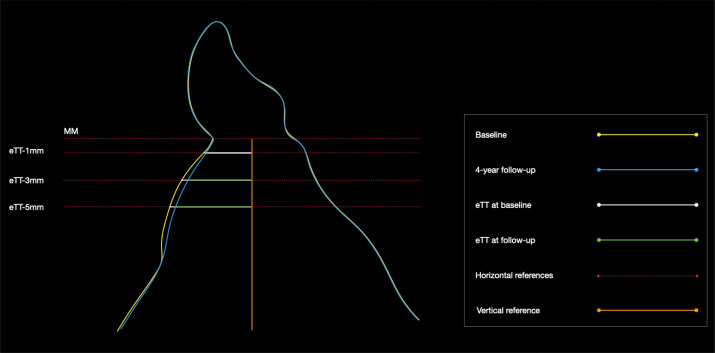
Schematic representation of reference lines and measurement points for linear tissue thickness analyses. The vertical reference line (orange) was positioned parallel to the long axis of the implant after alignment of central transversal images in the STL files. A horizontal reference line (red) was set at the level of the peri-implant mucosal margin (MM) at baseline. Additional horizontal lines were positioned 1, 3, and 5 mm apical to the MM (eTT-1 mm, eTT-3 mm, eTT-5 mm) for linear tissue thickness measurements. Baseline contours are shown in yellow, and 4-year follow-up contours in blue. Tissue thickness values at baseline (white lines) and at follow-up (green lines) were compared to assess dimensional changes over time.

### Patient-reported outcome measures (PROMs)

Patients’ quality of life was assessed through the 14-item Oral Health Impact Profile (OHIP-14) questionnaire. Additionally, overall satisfaction with treatment outcomes was self-reported by the patient using a Visual Analog Scale (VAS), ranging from 0 (“not satisfied at all”) to 100 (“completely satisfied”).

### Statistical analysis

Descriptive statistics were employed to summarize quantitative variables, with frequencies, mean, median, standard deviation (SD), and interquartile ranges reported. Shapiro-Wilk test was used to assess data normality, and Levene’s test was used to assess homoscedasticity prior to applying parametric or non-parametric tests. Comparisons between groups were made using the Fisher’s Exact test (for implant survival and implant success), Mann-Whitney U-test (for modified plaque index, mesial and distal bone levels, and patient-reported outcome measures) and Independent Samples t test (for keratinized tissue, distance between implant shoulder and mucosal margin, peri-implant attachment level, probing depth, PES, profilometric and linear measurements). Statistical significance was set at p < 0.05. As this was a non-interventional, follow-up study of an original non-inferiority randomized clinical trial, a sample size was not calculated.

## Results

### Demographics

From the original cohort of 64 patients who received lateral alveolar ridge augmentation, 20 agreed to participate in this follow-up study. The CXBB group included 11 patients (4 males and 7 females), with an average age of 51.3 years (SD 8.9). In the ABB group, there were 9 patients (4 males and 5 females), with an average age of 50.5 years (SD 9.4). There were no statistically significant differences in age or sex distribution between the groups.

### Survival and success rates

Early implant failures occurring prior to abutment connection were documented in a previous report. At the four-year follow-up, one late failure associated with parafunctional overloading was observed in the ABB group. There were no statistically significant differences in survival and success rates between the two groups (p < 0.05) (Table 1).

**Table 1:** Implant survival, success, and failures rates according with CXBB and ABB group.

ICOI Pisa Implant Quality of Health	CXBB (n = 11)	ABB (n = 9)
Late implant failure - n (%)	0 (0) A	1 (11.1) A
Success - n (%)	7 (63.6) A	7 (77.8) A
Satisfactory survival - n (%)	4 (36.4) A	1 (11.1) A
Compromised survival - n (%)	0 (0) A	0 (0) A
Failure - n (%)	0 (0) A	1 (11.1) A

CXBB: collagen xenogeneous bone block group; ABB: autogenous bone block group; ICOI: The International Congress of Oral Implantologists. Upper case letters represent intergroup comparison. Same letters indicate no significant difference (p ≥ 0.05) by Fisher’s Exact Test.

### Clinical and radiographic outcomes

At the four-year follow-up, no statistically significant differences were found between the CXBB and ABB groups regarding peri-implant clinical and radiographic outcomes (Table 2). Both groups demonstrated favorable clinical outcomes, characterized by low levels of plaque and BOP scores, along with consistently shallow PD. In all patients, the peri-implant mucosal margin remained coronal to the implant platform, indicating stable soft tissue support. Only two patients in each group presented with less than 2 mm of keratinized tissue. Mesial and distal peri-implant bone levels were similar between the groups.

**Table 2:** Clinical and radiographic parameters around implants in the CXBB and ABB grafted sites.

Clinical parameters	CXBB (n = 11)		ABB (n = 8)	
	Mean (SD)	Median (Q1:Q3)	Mean (SD)	Median (Q1:Q3)
mPI	0.6 (0.7) A	0.0 (0.0:1.7)	0.5 (0.7) A	0.0 (0.0:1.0)
BOP (%)	12.1 (29.9)	0 (0:16.6) A	6.2 (12.4)	0 (0:12.5) A
PD	2.2 (0.4) A	2.2 (1.8:2.5)	2.2 (0.9) A	1.9 (1.6:2.7)
MM	-1.8 (0.9) A	-1.8 (-1.0:-2.7)	-1.8 (0.7) A	-1.9 (-1.1:-2.4)
PAL	0.8 (0.6) A	1.0 (0.3:1.3)	1.2 (0.7) A	1.2 (0.5:1.8)
KT	2.4 (1.6) A	2.0 (1.0:3.0)	2.7 (1.8) A	3.0 (1.0:3.5)
BL mesial	0.14 (0.3)	0 (0.00:0.05) A	0.19 (0.41)	0 (0.00:0.25) A
BL distal	0.98 (1.15)	0.6 (0.00:2.15 ) A	0.88 (1.41)	0 (0.00:1.65) A

ABB: autogenous bone block group; BL: bone leve; BOP: bleeding on probing; CXBB: collagen xenogenous bone block group; dmPI: modified plaque index; KT: keratinized tissue; MM: distance between implant shoulder and mucosal margin; PAL: peri-implant attachment level; PD: probing depth; Q1: first quartile; Q3: third quartile; SD: standard deviation. Upper case letters represent intergroup comparison. Same letters indicate no significant difference (p ≥0 .05) by Mann-Whitney U-test or Independent Samples t Test.

### Pink esthetic score

At the four-year follow-up, the total PES score was 11.3 in the ABB group and 9.2 in the CXBB group, with no significant difference between the two groups (Table 3). Among the seven variables evaluated, only soft tissue level and contour demonstrated a statistically significant difference, with higher scores observed in the ABB group (Table 3).

**Table 3:** Pink Esthetic Scores for the soft tissues surrounding implants placed at sites grafted with CXBB and ABB.

Variable	CXBB (n = 11)		ABB (n = 8)	
	Mean (SD)	Median (Q1:Q3)	Mean (SD)	Median (Q1:Q3)
Mesial papilla	1.3 (0.6) A	1.0 (1.0:2.0)	1.7 (0.5) A	2.0 (1.0:2.0)
Distal papilla	1.2 (0.6) A	1.0 (1.0:2.0)	1.6 (0.5) A	2.0 (1.0:2.0)
Soft tissue level	1.2 (0.6) A	1.0 (1.0:2.0)	1.7 (0.5) B	2.0 (1.0:2.0)
Soft tissue contour	1.3 (0.5) A	1.0 (1.0:2.0)	1.9 (0.3) B	2.0 (2.0:2.0)
Soft tissue color	1.2 (0.7) A	2.0 (1.0:2.0)	1.6 (0.7) A	2.0 (1.0:2.0)
Alveolar process deficiency	1.6 (0.5) A	2.0 (1.0:2.0)	1.4 (0.5) A	1.0 (1.0:2.0)
Soft tissue texture	1.4 (0.7) A	1.5 (1.0:2.0)	1.6 (0.5) A	2.0 (1.0:2.0)
Total sum score	9.2 (2.7) A	9 (6.8:12.0)	11.3 (2.9) A	12.0 (8.0:14.0)

CXBB: collagen xenogenous bone block group; ABB: autogenous bone block group; SD: standard deviation; Q1: first quartile; Q3: third quartile. Upper case letters represent intergroup comparison. Different letters indicate significant difference (p < 0.05) by Independent Samples t Test.

### Profilometric and linear outcomes

The CXBB group had a mean overall tissue thickness gain of 0.89 (SD 0.64) mm, whereas the ABB group had a mean overall gain of 0.30 (SD 0.75) mm at 1 mm apical to the bone crest (Table 4). At 3 mm, The CXBB group had a mean overall tissue thickness gain of 0.35 (SD 0.96) mm, whereas the ABB group had a mean overall loss of 0.20 (SD 1.00) mm (Table 4).The mean volumetric gain in the CXBB group was 0.54 (SD 0.17) mm^2^, whereas the ABB group showed a mean gain of 0.57 (SD 0.43) mm^2^. These intergroup differences were not statistically significant (Table 4).

**Table 4:** Profilometric and linear measurements for CXBB and ABB.

Variable	CXBB (n = 11)		ABB (n=9)	
	Mean (SD)	Median (Q1:Q3)	Mean (SD)	Median (Q1:Q3)
4-year data				
eTT (1mm)	4.29 (0.90) A	3.98 (3.88:4.38)	3.66 (0.89) A	3.79 (3.44:4.27)
eTT (3mm)	4.82 (1.21) A	4.52 (4.16:4.80)	4.50 (1.22) A	4.94 (3.93:5.11)
eTT (5mm)	5.40 (1.49) A	5.00 (4.45:5.60)	5.05 (1.15) A	5,06 (4.47:5.58)
Difference (4 years - BL)				
eTT (1mm)	0.89 (0.64) A	1.15 (0.66:1.31)	0.30 (0.75) A	0.20 (0.00:0.71)
eTT (3mm)	0.35 (0.96) A	0.72 (-0.34:1.00)	-0.20 (1.00) A	0.10 (-0.65:0.44)
eTT (5mm)	-0.14 (1.11) A	0.00 (-0.62:0.22)	-0.31 (0.80) A	0.00 (0.00:0.00)
Profilometric changes (4 years - BL)	0.54 (0.17) A	0.54 (0.41:0.65)	0.57 (0.43) A	0.34 (0.26:0.96)

ABB: autogenous bone block group; BL: baseline; CXBB: collagen xenogenous bone block group; Q1: first quartile; Q3: third quartile; SD: standard deviation; eTT: estimated soft tissue thickness. Upper case letters represent intergroup comparison. Same letters indicate no significant difference (p ≥ 0.05) by Independent Samples t Test.

### Patient-reported outcome measures (PROMs)

While the CXBB group showed better results in the functional limitation domain, the other OHIP-14 domains and overall scores were comparable between the two groups (Table 5). Similarly, the VAS general satisfaction scores did not differ significantly between the CXBB and ABB groups (Table 5).

**Table 5:** Patient-reported outcome measures (PROMs) for CXBB and ABB group.

Variable	CXBB (n = 11)		ABB (n = 9)	
	Mean (SD)	Median (Q1:Q3)	Mean (SD)	Median (Q1:Q3)
OHIP-14 domains and total scores				
Functional limitation	0.0 (0.0)	0.0 (0.0:0.0) A	0.5 (1.0)	0.0 (0.0:0.0) B
Physical pain	0.4 (0.9)	0.0 (0.0:0.0) A	0.3 (0.8)	0.0 (0.0:0.0) A
Psychological discomfort	0.5 (1.1)	0.0 (0.0:0.0) A	0.7 (1.4)	0.0 (0.0:0.0) A
Physical disability	0.2 (0.7)	0.0 (0.0:0.0) A	0.5 (1.1)	0.0 (0.0:0.0) A
Physiological disability	0.3 (1.0)	0.0 (0.0:0.0) A	0.6 (1.3)	0.0 (0.0:0.0) A
Social disability	0.1 (0.3)	0.0 (0.0:0.0) A	0.1 (0.2)	0.0 (0.0:0.0) A
Handicap	0.0 (0.0)	0.0 (0.0:0.0) A	0.2 (0.9)	0.0 (0.0:0.0) A
Total sum score	**3.0 (6.4)**	**0.0 (0.0:1.0) A**	**5.6 (7.9)**	**2.0 (0.0:11.5) A**
Overall treatment satisfaction				
VAS Satisfaction	85.7 (19.6)	90 (90:100) A	94.0 (7.7)	100 (85:100) A

CXBB: collagen xenogenous bone block group; ABB: autogenous bone block group; SD: standard deviation; Q1: first quartile; Q3: third quartile. Upper case letters represent intergroup comparison. Same letters indicate no significant difference (p≥0.05) by Mann-Whitney U-test

## Discussion

The findings from this four-year follow-up study suggest that both CXBB and ABB are effective for staged lateral alveolar ridge augmentation, as demonstrated by the high survival and success rates of implants placed in CXBB and ABB augmented sites. Both CXBB and ABB grafted sites presented healthy peri-implant tissues and esthetic outcomes, with high patient satisfaction, reinforcing their long-term efficacy.

The implant survival and success rates observed at the four-year follow-up strongly support the long-term efficacy of both CXBB and ABB for staged lateral alveolar ridge augmentation. With only one late implant failure in the ABB group and none in the CXBB group, both grafting materials demonstrated adequate overall survival rates, consistent with our one-year follow-up findings ^26^ and corroborating other studies that report low late implant failure rates in CXBB- ^16,17,23^ and ABB-grafted sites.^31,32^ Furthermore, these results align with a recent systematic review and meta-analysis, which found no statistically significant difference in the survival rates of implants placed in CXBB and ABB-grafted sites (P = 0.71).^33^ The absence of significant differences in implant success rates between groups (63.3% for CXBB and 77.8% for ABB) further suggests the clinical reliability of CXBB as an alternative to ABB.

The high success rates observed in this study were consistently accompanied by clinical indicators of peri-implant health, highlighting the ability of both CXBB and ABB to maintain long-term peri-implant tissue stability over the 55-month post-loading follow-up period. Notably only one prior study has evaluated the long-term clinical outcomes of dental implants placed in CXBB-augmented sites over a similar duration.^17^ This specific case series,^17^ which followed 5 implants for 4.5-years, reported mean plaque index (PI = 0.5) and probing depth (PD = 2.96 mm) values closely matching those observed in the present study. Similarly, our results are in line with clinical data from implants placed in ABB-grafted sites and followed for at least five years.^34^ However, we observed higher BOP scores (12.1% for ABB and 6.2% for CXBB) compared to the lower BOP score (4.25%) reported by Schwarz and colaborators.^17^ This discrepancy may be attributed to differences in patient populations, oral hygiene practices, inflammatory response profiles, peri-implant soft tissue quality, and potentially differing maintenance protocols across the studies.^35^

In our research, PES for implants placed in CXBB-augmented sites averaged 9.2, whereas those placed in ABB-augmented sites averaged 11.3, reflecting high levels of esthetic acceptance and patient satisfaction. While overall outcomes were comparable between the CXBB and ABB groups, the ABB group exhibited superior soft tissue level and contour PES scores. Further investigation is warranted to determine whether the unique biological properties of autogenous bone, including its osteogenic, osteoinductive, and osteoconductive characteristics, may promote superior soft tissue integration and support, thereby contributing to more favorable esthetic outcomes. In contrast, CXBB grafts primarily promote new bone formation adjacent to the alveolar ridge, with a lesser degree of new bone formation and a higher prevalence of graft particles remaining lateral to the soft tissue,^20^ potentially limiting their ability to achieve the same degree of esthetic refinement as ABB. Additionally, the fact that sites augmented with ABB gained vertical bone height (0.38 mm), while those treated with CXBB experienced vertical bone loss (-0.16 mm),^25^ could further justify the differences observed in soft tissue level and contour. Further studies should explore the esthetic outcomes of peri-implant tissues in sites grafted with different bone substitute materials.

Profilometric and linear measurements revealed statistically comparable outcomes between CXBB and ABB-augmented sites, with both groups exhibiting minimal volumetric and thickness changes over the four-year follow-up. These findings corroborate previous research,^25^ reinforcing the capacity of both materials to effectively maintain the dimensional stability, which is critical for long-term peri-implant health and aesthetic success. However, the lack of statistically significant differences between CXBB and ABB groups might also be related to the limited sample size.

Patient-reported outcome measures (PROMs) are essential in assessing the success of dental implant therapies, as they provide insights into how treatments impact their quality of life. In this study, PROMs were evaluated using the OHIP-14 questionnaire and VAS satisfaction scores, revealing comparable outcomes between the CXBB and ABB groups, consistent with the results observed in the one-year follow-up.^26^ Notably, while CXBB group showed slightly better outcomes in the functional limitation domain of the OHIP-14, the ABB group also reported low scores in this domain, reinforcing the long-term suitability of both materials in maintaining high levels of patient satisfaction. Although post-surgical morbidity was notably high in the immediate postoperative period,^24^ these effects were transient and did not negatively impact long-term patient-reported outcomes after one ^26^and four years.

Despite the valuable insights gained from this study, some limitations should be acknowledged. Initially, 64 patients were treated from 2017-2020, but the follow-ups were significantly impacted in the aftermath of the COVID-19 pandemic, which significantly affected participants’ availability and willingness to return. Despite multiple contact attempts, many patients could not be reached, and among those who responded, relocation due to job or housing changes was the most frequently reported reason for non-participation, which reduced the cohort to a final sample size of 20 patients. This substantial loss to follow-up highlights a critical challenge in long-term clinical research, limiting the generalizability of the findings. Moreover, it may have reduced the statistical power to detect subtle differences between groups. Additionally, the study was conducted within a specific patient population, which may not fully represent the diversity of cases seen in clinical practice. Variations in systemic health, oral hygiene habits, and other patient-specific factors could have influenced the outcomes, but may not be fully accounted for here. Moreover, while the follow-up period was extended to four years, it may still be insufficient to capture all potential late implant failures or long-term complications, particularly those related to peri-implantitis. Lastly, although the present findings support the clinical reliability of CXBB as a viable alternative to ABB for lateral alveolar ridge augmentation, it is important to acknowledge that CXBB is currently not commercially available, which limits its immediate clinical applicability and reinforces the exploratory nature of this research. Nonetheless, the inclusion of CXBB in this clinical trial adds to the growing body of evidence regarding xenogeneic block graft materials and may guide future product development and clinical adoption.

Within the limitations of this study, particularly the reduced number of patients available for long-term follow-up, CXBB may be considered a feasible alternative to ABB for staged lateral alveolar ridge augmentation. Despite slightly better esthetic outcomes with ABB, clinical, radiographic and patient-reported outcomes were comparable between CXBB and ABB groups, reinforcing their reliability and warranting further investigation in larger cohorts.

## Data Availability

The datasets generated during and/or analyzed during the current study are available from the corresponding author on reasonable request.
